# The anti-tumor efficacy of 3-(2-Nitrophenyl) propionic acid-paclitaxel (NPPA-PTX): a novel paclitaxel bioreductive prodrug

**DOI:** 10.18632/oncotarget.10310

**Published:** 2016-06-27

**Authors:** Ping Song, Xin Yao, Ting Zhong, Shuang Zhang, Yang Guo, Wei Ren, Dan Huang, Xiao-Chuan Duan, Yi-Fan Yin, Shu-Shi Zhang, Xuan Zhang

**Affiliations:** ^1^ Beijing Key Laboratory of Molecular Pharmaceutics and New Drug Delivery Systems, School of Pharmaceutical Sciences, Peking University, Beijing 100191, China; ^2^ Department of Pharmaceutics, School of Pharmaceutical Sciences, Peking University, Beijing 100191, China

**Keywords:** paclitaxel, 3-(2-Nitrophenyl) propionic acid, bioreductive prodrug, tumor hypoxia, anti-tumor efficacy

## Abstract

Hypoxia is an important microenvironmental pressure present in the majority of solid tumors and, so, tumor hypoxia might be considered an attractive target for tumor therapy. One strategy for targeting hypoxia is to develop bioreductive prodrugs. In the present research, we synthesized a bioreductive paclitaxel prodrug, 3-(2-Nitrophenyl) propionic acid-paclitaxel (NPPA-PTX). The stability of NPPA-PTX in PBS and rat plasma was investigated. The anti-tumor activity of NPPA-PTX was also evaluated *in vitro* and *in vivo*. The results of our stability study indicated that NPPA-PTX was stable in PBS and rat plasma as well as in the blood circulation. The *in vitro* and *in vivo* anti-tumor activity of NPPA-PTX was confirmed in both KB cells and MDA-MB-231 cells. Our results also indicated that NPPA-PTX could completely convert to active PTX in tumor tissues and produced the anti-tumor activity in both KB and MDA-MB-231 tumor-bearing nude mice. We suggest that the dissociated PTX which converted from NPPA-PTX in tumor tissues played a key role in producing anti-tumor activity. Considering all our results, we suggest that NPPA-PTX is a novel bioreductive PTX prodrug which could undergo further evaluation.

## INTRODUCTION

Hypoxia is a common feature of most tumors because of the limited oxygen diffusion and highly abnormal tumor microvasculature [1–3]. It is a negative factor owing to its multiple contributions to a variety of phenomena such as chemo/radio-resistance, angiogenesis, and metastasis. Also, hypoxic tumor cells exhibit a greater capacity for reductive reactions compared with normal well-oxygenated cells [4–5]. Therefore, tumor hypoxia is an attractive target for tumor therapy [1]. One strategy for targeting hypoxia is to develop bioreductive prodrug that is activated by enzymatic reduction in hypoxic tissue [6–7]. To date, five distinct types of bioreactive prodrugs, including nitro groups, quinones, aromatic N-oxides, aliphatic N-oxides and transition metals, have been developed for targeting hypoxic cells in solid tumors [1]. Several examples including PR-104, TH-302, and EO9 are reported to be undergoing phase II/III clinical evaluation [8–9].

Nitrophenylalkanoic acid is reported to target hypoxic region of solid tumors as shown in early radio therapeutic enhancement *in vivo* [10]. Its derivatives tend to be nontoxic or only weakly toxic in normal tissues but become activated in hypoxic tumor tissues [11–12]. Introduction of nitrophenylalkanoic acid to form conjugate with conventional chemotherapeutic drug is relatively simple and the produced prodrug tends to exhibit specific selectivity for hypoxic tumor tissues [13–14]. The nitro group of the prodrug is assumed to be reduced under reducing conditions, thereby undergoing subsequent activation through intramolecular cyclization to release the parent drug [15]. In addition, some research has proposed that the activation process tends to be triggered in an acidic environment, which suggests that it is more likely to be reduced at a low pH in tumor tissues [16].

Paclitaxel (PTX) is one of the most successful anticancer drugs in clinical use, and it exhibits high anti-tumor efficacy against a wide range of tumors [17–18]. As the free 2′-hydroxyl group is an important active part of PTX structure, it is easier to introduce other groups at the 2′-position to obtain a prodrug with little or no potency in the normal internal environment while being activated in tumor tissues [19]. There are many examples of the use of this chosen strategy, but they could not achieve valid release at the disease site or were released too early in circulation [20–21].

In the present study, we conjugated PTX with 3-(2-nitrophenyl)propionic acid (NPPA) at the 2′-hydroxyl group of PTX to produce a novel bioreductive PTX prodrug (NPPA-PTX) which could remain intact and stable in normoxia but be activated in hypoxic tumor tissues. The anti-tumor activity of NPPA-PTX was investigated *in vitro* and *in vivo*.

## RESULTS

**Figure 1:**
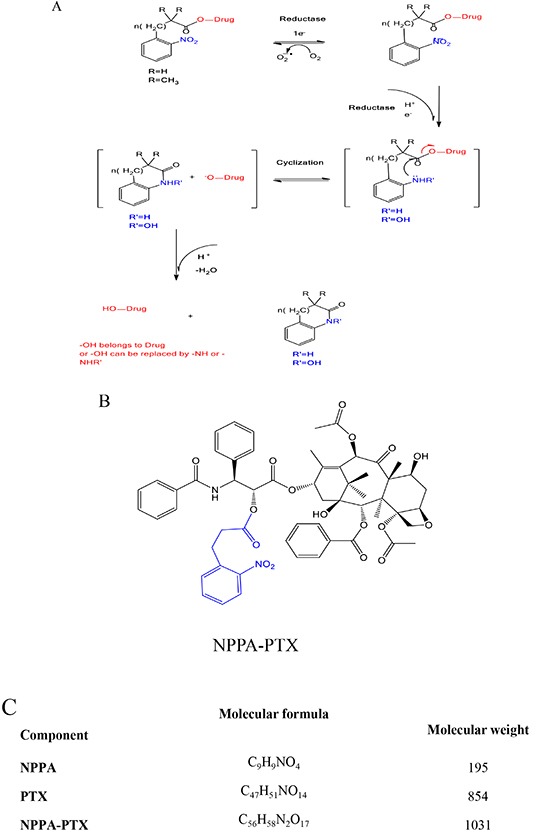
Proposed activation mechanism of nitrophenylalkanoic acid bioreductive prodrugs under convertible reduction **(A).** Structures and molecular formulas of NPPA-PTX **(B** and **C)**.

As shown in Figure 1A, the nitro group of nitrophenylalkanoic acid bioreductive prodrugs may undergo a convertible reduction via 1e^−^ addition in the presence of oxygen, but an irreversible reduction occurred without oxygen. Then, these bioreductive prodrugs continue to be reduced gradually into hydroxy-amine or amino derivatives, thereby releasing PTX through intramolecular cyclization. The structure and molecular formula of NPPA-PTX are shown in Figure 1B and 1C. Our MALDI-TOF spectrum of NPPA-PTX indicate that PTX was successfully conjugated with NPPA (data not shown).

**Figure 2:**
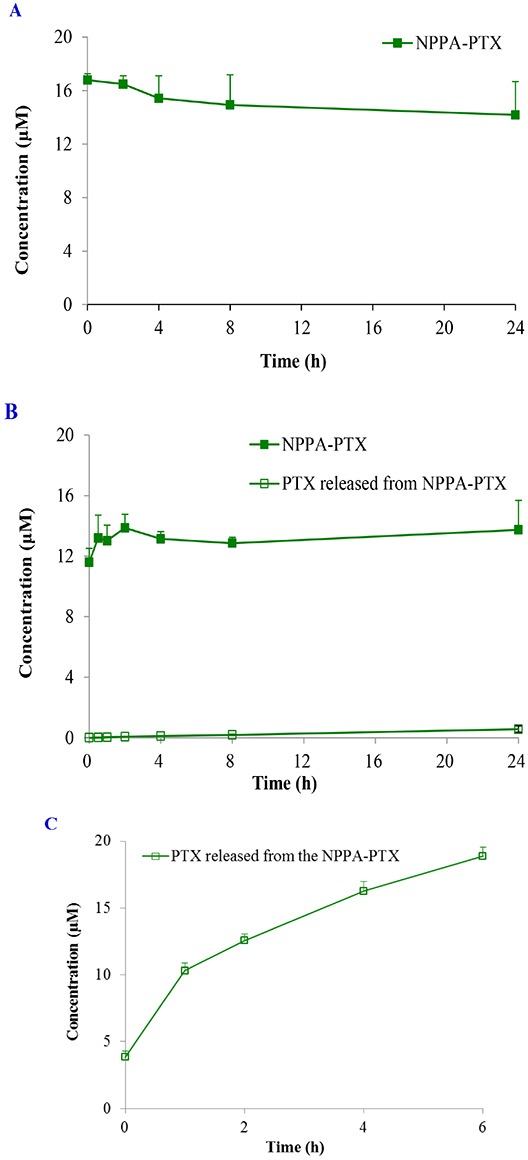
The stability of NPPA-PTX in PBS, rat plasma or incubated with nitroreductase The stability of NPPA-PTX in PBS at 37°C. **(A);** in rat plasma at 37°C **(B);** incubated with nitroreductase at 37°C **(C)**.

Because the ester linkage in the NPPA-PTX molecule might be susceptible to hydrolysis, the stability of NPPA-PTX is an important issue. The *in vitro* stability of NPPA-PTX was investigated in PBS and rat plasma. Our results indicated that NPPA-PTX was stable in PBS solution for at least 24 hours without dissociation of PTX from the NPPA-PTX, as shown in Figure 2A. Our results also indicated that NPPA-PTX was also stable in rat plasma at least 24 hours with less than 4.2 % dissociated PTX (Figure 2B). The enzymolysis of NPPA-PTX was evaluated *in vitro*. As shown in Figure 2C, we found the dissociated PTX released from NPPA-PTX.

**Figure 3:**
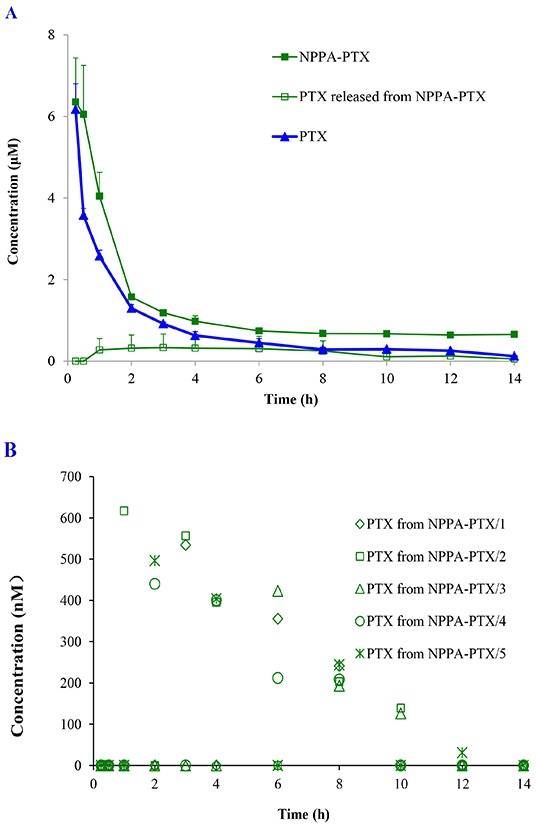
Plasma concentration profiles of NPPA-PTX, dissociated PTX released from NPPA-PTX and PTX Plasma concentration profiles of NPPA-PTX, dissociated PTX released from NPPA-PTX and PTX after a single intravenous administration of NPPA-PTX at 6.04 mg/kg (equimolar with 5 mg/kg PTX) or Taxol (PTX 5mg/kg) in rats (n = 5). **(A).** The plasma concentrations of dissociated PTX released from NPPA-PTX in the individuals rat after i.v. injection in rats (n = 5) **(B).**

The concentration of NPPA-PTX in plasma was determined after a single intravenous injection of NPPA-PTX at a dose equimolar with 5 mg/kg PTX to SD rats *in vivo*. We observed that the dissociated PTX released from NPPA-PTX was much lower than that of NPPA-PTX, as shown in Figure 3A, indicating that NPPA-PTX was stable in the blood circulation and might be distributed to the tissues as the intact NPPA-PTX. In fact, the dissociated PTX released from the NPPA-PTX could only be detected occasionally in a couple of rats, which also showed that the NPPA-PTX remained structurally intact in the blood circulation (Figure 3B). As shown in Figure 3A, the most concentration points of NPPA-PTX in plasma were higher than that of PTX. The typical pharmacokinetic parameters of NPPA-PTX and PTX are summarized in Table 1. As shown in Table 1, the values of AUC_0-14_, AUC_0-inf_, t_1/2_, MRT_0-14_ and MRT_0-inf_ in the NPPA-PTX group were significant higher than that in the Taxol group (*p<0.01*). As prolonged blood circulation is the driving force for increased tumor targeting, the NPPA-PTX was supposed to show an improved therapeutic efficacy.

**Table 1:** The main pharmacokinetic parameters of NPPA-PTX and PTX after intravenous administration of the NPPAPTX (equimolar with 5 mg/kg PTX) and Taxol (PTX 5 mg/kg) in rats (mean ± S.D., n = 5)

Parameters	Unit	PTX	NPPA-PTX
C_max_	μM	6.2±0.6	6.7±0.8
AUC_(0-14)_	μM*h	6.0±0.5	18.5±1.5**
AUC_0-inf_	μM*h	6.6±0.4	40.1±9.8**
t_1/2_	h	4.6±1.0	24.4±9.6**
MRT_(0-14)_	h	3.0±0.2	3.9±0.2**
MRT_0-inf_	h	4.9±0.5	28.6±11.4**

** *p<0.01 vs* PTX group

**Figure 4:**
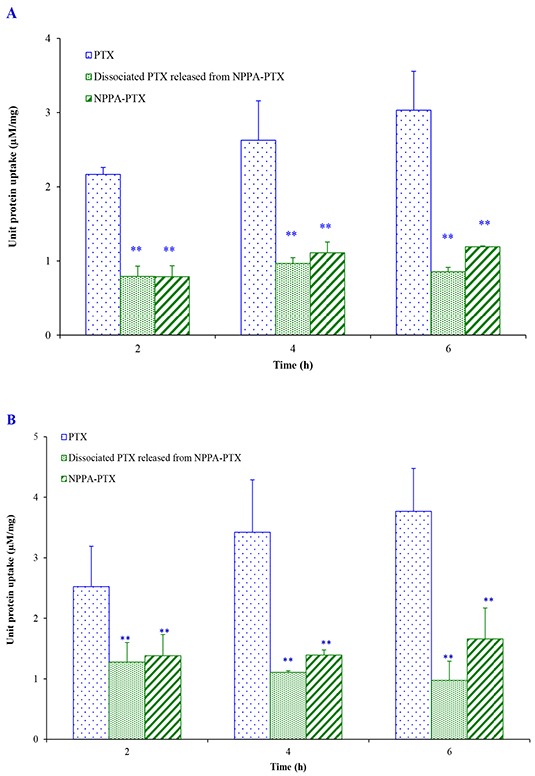
Cell uptake efficiency following culture with PTX and NPPA-PTX for 2, 4, 6 h in KB cells **(A)** and MDA-MB-231 cells **(B).** ** *p*<0.01, compared with PTX group.

*In vitro* cellular uptake of NPPA-PTX was examined in the KB and MDA-MB-231 cell lines. As shown in Figure 4A, NPPA-PTX exhibited decreased cellular level in KB cells compared with free PTX after an incubation of 2, 4, and 6 h (*p*<0.01). However, the dissociated PTX released from NPPA-PTX was also observed, indicating that the NPPA-PTX could dissociate to active PTX. Similar results were also shown in MDA-MB-231cells (Figure 4B).

The *in vitro* anti-tumor activity of NPPA-PTX in KB and MDA-MB-231 cells was also evaluated. In the KB cell line, the calculated IC_50_ value was found to be 0.33 ± 0.05 μM for NPPA-PTX, which was significantly higher than that of free PTX (0.16 ± 0.04 μM) (*p*<0.01), as shown in Table 2. Similar results were also observed in MDA-MB-231 cell line.

**Table 2:** IC50 values (μM) of NPPA-PTX on KB and MDA-MB-231 cell lines in normal and simulative hypoxic condition

Cell lines	Normal condition		simulative hypoxic condition	
	PTX	NPPA-PTX	PTX	NPPA-PTX
KB	0.155±0.035	0.331±0.048 **	0.127±0.014	0.209±0.033**$$
MDA-MB-231	1.36±0.21	3.78±0.32 **	1.118±0.207	1.830±0.260**$$

** *p*<0.01, compared with PTX group.

$$ *p*<0.01, compared with normal condition.

The *in vitro* anti-tumor activity of NPPA-PTX in KB and MDA-MB-231 cells was also evaluated in the simulative hypoxic condition. In the KB cell line, the calculated IC_50_ value was found to be 0.209±0.033 μM for NPPA-PTX, which was significantly higher than that of free PTX (0.127±0.014 μM) (*p*<0.01), as shown in Table 2. Similar results were also observed in MDA-MB-231 cell line.

In the simulative hypoxic condition, the IC_50_ values of NPPA-PTX in KB or MDA-MB-231 cell lines, unlike PTX, were significant lower than that in normal condition, indicating the reduction of NPPA-PTX in the simulative hypoxic condition, as shown in Table 2.

**Figure 5:**
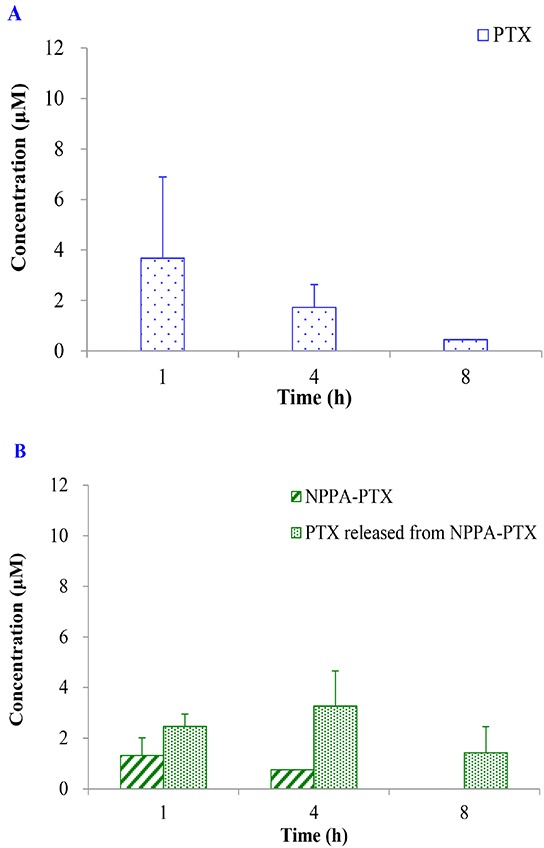

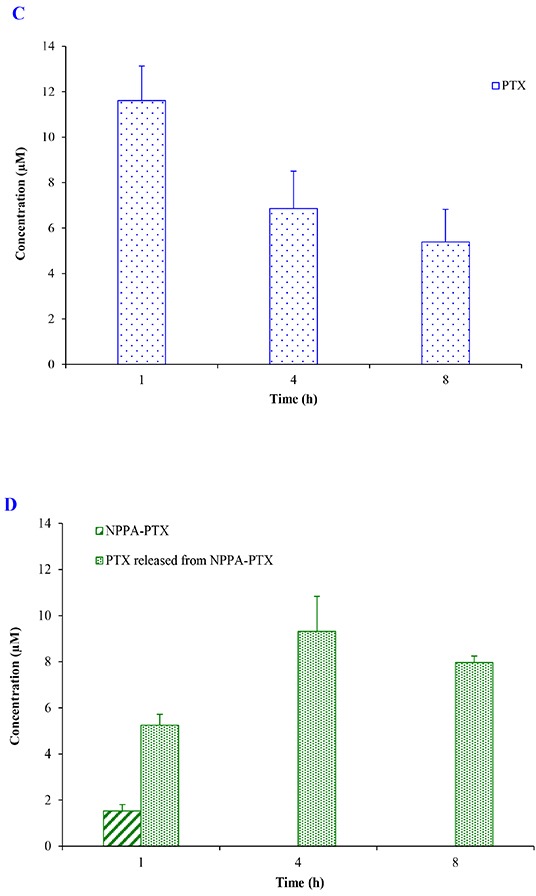
The PTX, NPPA-PTX and PTX released from NPPA-PTX levels (μM) in tumor tissues after a single intravenous administration of Taxol at a dose of 15 mg/kg or NPPA-PTX at a dose of 18.11 mg/kg (equimolar with 15 mg/kg PTX) to KB (A and B) MDA-MB-231 (C and D) tumor-bearing nude mice (Mean±SD and n=3).

The dissociated PTX from NPPA-PTX in tumor tissues was detected after a single intravenous injection of NPPA-PTX at a dose equimolar with 15 mg/kg PTX to KB or MDA-MB-231 tumor-bearing nude mice, respectively. As shown in Figure 5B, the dissociated PTX in the KB tumor tissues was detected at 1 h as well as 4 and 8 h. Compared with the free PTX in KB tumor tissues in the Taxol treatment group (Figure 5A), the dissociated PTX in the NPPA-PTX treatment group was 0.7-, 1.9- and 3.2-fold compared with that in Taxol treatment group at 1, 4 and 8 h. We also examined the level of NPPA-PTX in KB tumor tissues after administration of NPPA-PTX (Figure 5B). Compared with the free PTX in tumor tissues in the Taxol treatment group, the level of NPPA-PTX was significantly lower than that of free PTX. At 8 h, no NPPA-PTX was detected in the KB tumor tissues, indicating that NPPA-PTX was completely transformed into dissociated PTX. Similar results were observed in MDA-MB-231 tumor-bearing nude mice tumor tissues (Figure 5C and 5D). The dissociated PTX in the NPPA-PTX treatment group was 0.5-, 1.4- and 1.5-fold compared with that in Taxol treatment group at 1, 4 and 8 h. At 4 and 8 h, no NPPA-PTX was detected in the MDA-MB-231 tumor tissues at 4 and 8 h time points, also indicating that NPPA-PTX was completely transformed into dissociated PTX.

**Figure 6:**
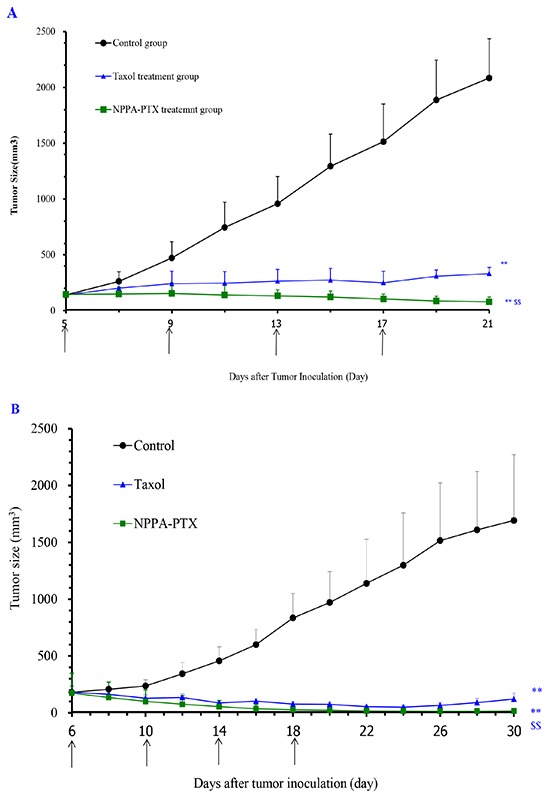
*In vivo* anti-tumor activity of NPPA-PTX in KB (A) and MDA-MB-231 (B) tumor-bearing nude mice KB cells were implanted in the nude mice on the 0 day, and the treatment was started on the 5th day when the tumor volume reached about 100-200 mm^3^. The treatment involved administration of saline solution, Taxol and NPPA-PTX on the 5th, 9th, 13rd and 17th day, respectively. Data are presented as the mean ± SD per group measured at indicated days after treatment (n=6). MDA-MB-231 cells were implanted in the nude mice on the 0 day, and the treatment was started on the 6th day when the tumor volume reached about 100-200 mm^3^. The treatment involved administration of saline solution, Taxol and NPPA-PTX on the 6th, 10th, 14th and 18th day, respectively. Data are presented as the mean ± SD per group measured at indicated days after treatment (n=6). ** *p*<0.01, compared with the control group; $$ *p*<0.01, compared with the Taxol treatment group.

The *in vivo* anti-tumor activity of NPPA-PTX was evaluated in KB and MDA-MB-231 tumor-bearing nude mice, respectively. As shown in Figure 6A, the tumor growth was significantly inhibited in Taxol and NPPA-PTX treatment groups compared with the physiological saline treatment group (*p*<0.01). Interestingly, NPPA-PTX significantly inhibited the growth of KB tumors compared with that in the PTX treatment groups (*p*<0.01). The average tumor size at day 21 in the PTX and NPPA-PTX treatment groups was 331±56 mm^3^ and 78±43mm^3^, respectively, compared with 2085 ± 353 mm^3^ in the physiological saline group (*p*<0.01). The corresponding tumor growth inhibition in the PTX and NPPA-PTX -treated groups was about 84 % and 96 %.

Similar results were also observed in MDA-MB-231 tumor-bearing nude mice, as shown in Figure 6B. The average tumor size at day 30 in the PTX and NPPA-PTX treatment groups was 121±52 mm^3^ and 17±23mm^3^, respectively, compared with 1692 ± 579 mm^3^ in the physiological saline group (*p*<0.01). The corresponding tumor growth inhibition in the PTX and NPPA-PTX -treated groups was about 93 % and 99 %.

## DISCUSSION

The hypoxic cells could limit the activity of many chemotherapeutic drugs. Nontoxic prodrugs that generate active species in hypoxic tissue by selective bioreduction have long been explored [22].

PTX is one of the most powerful anti-cancer agents currently used in clinic situations. In order to target hypoxic cells in tumors, many bioreductive PTX prodrugs have been investigated [23–26]. These prodrugs were constructed in a manner in which reductive activation, catalyzed by reductive endogenous enzymes, led to PTX release. However, there are few reports about the *in vivo* anti-tumor activity and bio-distribution in tumor tissues of bioreductive prodrugs. Recently, a PTX bioreductive prodrug which was activated specifically by prostate-specific antigen has been reported [27]. This bioreductive prodrug could be cleaved rapidly releasing the PTX-dipeptide, and then degraded to liberate PTX as a final cleavage product within a few hours in prostate tumor tissue. However, the synthetic process of this bioreductive prodrug is more complex and the anti-tumor activity of this bioreative prodrug is comparable with PTX.

It has been reported that nitrophenylacetic acid compounds can be used as triggers for producing bioreductive prodrugs [10, 12, 28–29]. In the present research, we synthesized a novel bioreductive PTX product NPPA-PTX. Our stability results indicated that NPPA-PTX was stable in PBS and rat plasma as well as in the blood circulation.

In the cellular uptake experiment, we observed that NPPA-PTX could partly convert to active parent drug in both KB and MDA-231 cells. We suggested that the *in vitro* anti-tumor activity of NPPA-PTX was produced by dissociated PTX. In the *in vitro* simulative hypoxic condition, the anti-tumor activity of NPPA-PTX in KB or MDA-MB-231 cell lines, unlike PTX, were significant higher than that in normal condition, indicating the reduction of NPPA-PTX in the simulative hypoxic condition.

Interestedly, NPPA-PTX was completely converted to active PTX in both KB and MDA-MB-231 tumor tissues. This indicated that the hypoxic microenvironment in the tumor region is one of the most important factors for NNPA-PTX being converted to the parent drug. Because of the concentration of dissociated PTX significantly higher than that of free PTX in both KB and MDA-MB-231 tumor tissues, we suggested that the *in vivo* anti-tumor activity of NPPA-PTX which produced by the dissociated PTX would be higher than that of free PTX in both KB and MDA-MB-231 tumor bearing mice. In addition, our pharmacokinetic results also indicated that NPPA-PTX has a prolonged blood circulation than that of PTX, showing the potential improved therapeutic efficacy. Therefore, we believe that NPPA-PTX was a more effective PTX bioreductive prodrug which could be used for further evaluation.

In summary, we synthesized a novel bioreductive PTX prodrug NPPA-PTX. Our stability results indicated that NPPA-PTX was stable in PBS and rat plasma as well as in the blood circulation. The *in vitro* and *in vivo* anti-tumor activity of NPPA-PTX was confirmed in both KB cells and MDA-MB-231 cells. Our results also indicated that NPPA-PTX could completely convert to active PTX in tumor tissues and produced the anti-tumor activity in both KB and MDA-MB-231 tumor-bearing nude mice. We believe that the dissociated PTX which converted from NPPA-PTX in tumor tissues played a key role in producing anti-tumor activity. Considering all our results, we suggest that NPPA-PTX is a novel bioreductive PTX prodrug which could undergo further evaluation.

## MATERIALS AND METHODS

### Materials

3-(2-Nitrophenyl) propionic acid (NPPA) was obtained from Beijing OUHE Tech. Co. LTD (Beijing, China). N, N'-Dicyclohexylcarbodiimide (DCC), 4-Dime-thylaminopyridine (DMAP) and SRB were all purchased from Sigma-Aldrich (St. Louis, MO, USA). Paclitaxel (PTX) was obtained from Mei-Lian Co., Ltd. (Chongqing, China). Cremophor EL (CrEL) was purchased from BASF Corporation of Germany (Local agent in Shanghai, China). Paclitaxel injection (Taxol) was obtained from a local hospital in Beijing (Bristol Myers Squibb Co., Princeton, NJ, USA). Cell culture media RPMI 1640, penicillin streptomycin, fetal bovine serum, and L-glutamine were obtained from GIBCO, Invitrogen Corp. (Carlsbad, California, USA). Methylene chloride was dehydrated and distilled immediately before use. All other chemicals were of analytical or HPLC grade.

### Cell lines

The KB human mouth oral squamous cell carcinoma (OSSC) cell line was obtained from the Chinese Academy of Sciences Cells Bank (Shanghai, China) and cultivated in RPMI-1640 medium. Each cell culture medium was supplemented with 10 % FBS, 100 units/ml penicillin and 100 μg/ml streptomycin. The cultures were maintained at 37°C, 95 % relative humidity and 5 % CO_2_.

The MDA-MB-231 cell lines, a human breast cancer cells (ER negative), was obtained from the Chinese Academy of Sciences Cells Bank (Shanghai, China) and cultivated in L-15 medium which was supplemented with 10 % FBS, 100 units/ml penicillin and 100 μg/ml streptomycin. The cultures were maintained at 37°C, 95 % relative humidity.

### Synthesis of NPPA-PTX

Briefly, PTX (70 mg, 82 mmol) was dissolved in anhydrous methylene chloride (5 ml), and then NPPA (10 mg, 82mmol) and DCC (33.8 mg, 164 mmol) were added. The reaction mixture was stirred at room temperature for 24 h. The residue was evaporated under nitrogen and then separated by thin-layer chromatography using methylene chloride/methanol (20:1) as the developing solvent. The silica gel which adsorbed the target product was collected and shaken with ethyl acetate then dried under vacuum to obtain the final product (white powder). The purity of NPPA-PTX was 99.7% (analyzed by HPLC). The structure of NPPA-PTX was characterized by mass spectrometry (MALDI-TOF-MS instrument).

### Stability of NPPA-PTX in PBS and rat plasma

The NPPA-PTX was dissolved in PBS (pH 7.4) at a final concentration of 20 μM and incubated at 37°C with gentle shaking. Similarly, NPPA-PTX PBS solution was added to rat plasma (the final concentration was 20 μg/ml) and incubated at 37°C with gentle shaking. At scheduled time, samples were analyzed by HPLC as described below to determine the released free PTX. Each experiment was carried out in triplicate.

### Nitroreductase assay

NPPA-PTX was dissolved in 50 mM Tris-Cl (pH 7.0) buffer, filtered and degassed for 5 min, to give a final concentration of 1 mM. The reaction was carried out anaerobically by repetitive bubbling with nitrogen for 10 min before and after the addition of nitroreductase. To this solution, nitroreductase and NADPH were added to give final concentrations of 50 μg/ml and 3.4 mM, respectively, and incubated at 37°C. At scheduled times, samples were taken and the reaction stopped by the addition of ice-cold methanol, and then analyzed by HPLC after being centrifuged at 6000 g for 5 min. Each experiment was carried out in triplicate.

### Stability of NPPA-PTX in rat blood circulation

SD rats weighing 200±20 g were allowed free accessing to standard food and water, maintained on a light/dark cycle under conditions of 25±3°C and 50 % humidity, and allowed to acclimatize for 7 days. All care and handling of animals were performed with the approval of the Institutional Authority for Laboratory Animal Care of Peking University.

The NPPA-PTX solution was prepared using CrEL/ethanol/saline (1:1:8 v/v/v) as a solvent. Then, NPPA-PTX solution was injected intravenously into rats at a dose of 6.04 mg/kg (equimolar with 5 mg/kg PTX). In addition, Taxol was injected into the tail vein at a single dose of 5 mg/kg. Blood samples (0.5 ml) were collected via the orbital venous plexus at 0.25, 0.5, 1, 2, 4, 6, 8, 10, 12, 14 h after injection. After centrifugation at 6000 g for 5 min, the obtained plasma was stored at −20°C until required for HPLC analysis.

Pharmacokinetic parameters were calculated from PTX and NPPA-PTX concentration-time data using noncompartmental methods as implemented by the program WinNonlin version 3.1 (Pharsight Corp., Mountain View, CA, USA). C_max_ was the observed values. The AUC was calculated using the linear trapezoidal method and was extrapolated to infinity (AUC_inf_) by dividing the last measured concentration by the terminal rate constant, λ_z_, which was determined from the slope of the terminal phase of the plasma concentration-time curve. The terminal half-life (t_1/2_) was calculated as 0.693 divided by λ_z_.

### *In vitro* cellular uptake

KB cells were seeded in a 6-well flat-bottom tissue-culture plate at a density of 3×10^5^ cells/well with 2 ml growth medium. After 24 h, the medium was replaced with NPPA-PTX solution (10 μM), and incubated for 2, 4 or 6 h at 37°C. After incubation, cells were washed twice with cold PBS to remove unbound drug followed by the addition of 200 μl 10 % SDS solution, then 10 μl samples were taken for determination of the protein concentration. Following this, the samples were vortexed for 1 min after addition of 300 μl acetonitrile for protein precipitation. A volume of 30 μl of supernatant was used for HPLC detection after centrifugation at 6000 g for 5 min [30]. For each sample, three wells were measured.

Similarly, MDA-MB-231 cells were seeded respectively in a 6-well flat-bottom tissue-culture plate at a density of 5×10^5^ cells/well with 2 ml growth medium, and then proceed as above procedure.

### *In vitro* cytotoxicity

The *in vitro* cytotoxicity of NPPA-PTX against KB cells and MDA-MB-231 cells was measured using the SRB method. The KB cells (8×10^3^ cells/well) and MDA-MB-231 cells (1×10^4^ cells/well) were seeded in 96-well plates and incubated for 24 hours, respectively. After that, the cells were treated with different concentrations of NPPA-PTX and incubated for 48 h at 37°C. The cell viability was determined by sulforhodamine B assay. Absorbance was measured at 540 nm using a 96-well plate reader (model 680; Bio-Rad Laboratories, Hercules, CA, USA). The survival percentages were calculated using the formula: survival % = (A540 nm for the treated cells/A540 nm for the control cells) ×100%, where A540 nm is the absorbance value. Each assay was carried out in triplicate. Finally, dose-effect curves were constructed and IC_50_ values were calculated.

The *in vitro* cytotoxicity of NPPA-PTX in simulative hypoxic condition was also investigated. As same as the above procedure, the cell lines were cultured in simulative hypoxic condition with a modular incubator (MIC 101) and the dual flow meters (one flow is 5% CO_2_ and the other flow is 5% O_2_ mixed with 90% CO_2_.) (Billups-Rothenberg, inc. (California, USA)).

### Distribution in tumor tissues

The distribution of NPPA-PTX in tumor tissues was evaluated in KB and MDA-MB-231 tumor-bearing nude mice. For preparation of the tumor-bearing KB and MDA-MB-231 mice model, briefly, BALB/c nude mice were subcutaneously injected in the right flank with 0.2 ml cell suspension containing 3×10^6^ KB cells or 1×10^7^ MDA-MB-231 cells with 20 % basement membrane matrix, respectively. The BALB/c mice were randomly divided into 2 treatment groups (9 mice per group), and the tumors were allowed to grow for 7 days to a volume of 400 mm^3^. Then, each group was given a tail vein injection of Taxol (15 mg/kg PTX) or NPPA-PTX (18.10 mg/kg, equimolar with 15 mg/kg PTX). Mice were executed at 1, 4 and 8 h (each group containing three mice). The tumor tissues were removed and blotted with a paper towel, rinsed in saline, then blotted to remove excess fluid, weighed and stored at −20°C until required for analysis. The tumor tissues were weighed (1g) and homogenized with 3 ml PBS, followed by extraction and analysis as described for blood samples.

### *In vivo* anti-tumor efficacy

The *in vivo* anti-tumor efficacy of NPPA-PTX was evaluated in KB tumor-bearing nude mice. The BALB/c mice were randomly divided into three groups (6 mice per group), and the tumors were allowed to grow for 5 days to a volume of 100-200 mm^3^. Then, mice were given an intravenous injection of physiological saline, Taxol or NPPA-PTX at a dose of 15 or 18.10 mg/kg (equimolar with 15 mg/kg PTX, q3d×4) through the tail vein. The tumor volume was measured every two days using a calculation based on the equation (a×b^2^)/2, where a and b are the length and width of the tumor, respectively. The animals were also weighed every two days during the experimental period. After 21 days, all the mice were sacrificed, and the tumor tissues were removed and weighed.

The *in vivo* anti-tumor efficacy of NPPA-PTX was also evaluated in MDA-MB-231 tumor-bearing nude mice. The BALB/c mice were randomly divided into three groups (6 mice per group), and the tumors were allowed to grow for 6 days to a volume of 100-200 mm^3^. Then, mice were given an intravenous injection of physiological saline, Taxol or NPPA-PTX at a dose of 15 or 18.10 mg/kg (equimolar with 15 mg/kg PTX, q3d×4) through the tail vein. The tumor volume was measured every two days using a calculation based on the equation (a×b^2^)/2, where a and b are the length and width of the tumor, respectively. The animals were also weighed every two days during the experimental period. After 30 days, all the mice were sacrificed, and the tumor tissues were removed and weighed.

### HPLC analysis

NPPA-PTX in plasma was extracted by the following published method with minor modifications [30]. Briefly, plasma (100 μl) was mixed with 2.5 ml acetonitrile in a vortex mixer for 1 min. The mixture was then centrifuged at 6000 g for 5 min, and then 2.0 ml of supernatant was collected, and dried under a gentle stream of nitrogen at 50°C in a water bath. The residue was dissolved in 100 μl mobile phase by vortexing and the supernatant (30 μl) was injected into the HPLC system. The HPLC system consisted of a Waters 2487 Dual λ Absorbance Detector and 1525 pump. An ODS 3C-18 analytical column (5 μm, 250×4.6 mm, Phenomenex) was used and the wavelength was set at 227 nm. The mobile phase (acetonitrile : water 60:40 v/v) was pumped at a flow rate of 1 ml/min for 30 min. The retention time of PTX or NPPA-PTX was approximately 7 or 19 min respectively.

### Statistical analysis

All data are shown as means ± standard deviation (SD) unless stated otherwise. One-way analysis of variance (ANOVA) was used to determine significance among groups, after which post-hoc tests with the Bonferroni correction were used for comparisons between individual groups. Statistical significance was established at *p* < 0.05.
